# Design of Graphene Phononic Crystals for Heat Phonon Engineering

**DOI:** 10.3390/mi11070655

**Published:** 2020-06-30

**Authors:** Haque Mayeesha Masrura, Afsal Kareekunnan, Fayong Liu, Sankar Ganesh Ramaraj, Günter Ellrott, Ahmmed M. M. Hammam, Manoharan Muruganathan, Hiroshi Mizuta

**Affiliations:** 1 School of Material Science, Japan Advanced Institute of Science and Technology, Nomi 923-1211, Japan; s1620426@jaist.ac.jp (H.M.M.); afsal@jaist.ac.jp (A.K.); fayong@jaist.ac.jp (F.L.); ramaraj@jaist.ac.jp (S.G.R.); s1822014@jaist.ac.jp (G.E.); a-hammam@jaist.ac.jp (A.M.M.H.); mizuta@jaist.ac.jp (H.M.); 2 Physics Department, Faculty of Science, Minia University, 11432 Main Road—Shalaby Land, Minia 61519, Egypt; 3 Hitachi Cambridge Laboratory, Hitachi Europe Ltd., Cavendish Laboratory, JJ Thomson Avenue, Cambridge CB3 0HE, UK

**Keywords:** graphene nanomesh, phononic bandgap, Finite Element Method (FEM) simulation, circle-cross-snowflake shaped nanopores, heat phonon engineering, graphene phononic crystals

## Abstract

Controlling the heat transport and thermal conductivity through a material is of prime importance for thermoelectric applications. Phononic crystals, which are a nanostructured array of specially designed pores, can suppress heat transportation owing to the phonon wave interference, resulting in bandgap formation in their band structure. To control heat phonon propagation in thermoelectric devices, phononic crystals with a bandgap in the THz regime are desirable. In this study, we carried out simulation on snowflake shaped phononic crystal and obtained several phononic bandgaps in the THz regime, with the highest being at ≈2 THz. The phononic bandgap position and the width of the bandgap were found to be tunable by varying the neck-length of the snowflake structure. A unique bandgap map computed by varying the neck-length continuously provides enormous amounts of information as to the size and position of the phononic bandgap for various pore dimensions. We have also carried out transmission spectrum analysis and found good agreement with the band structure calculations. The pressure map visualized at various frequencies validates the effectiveness of snowflake shaped nano-pores in suppressing the phonons partially or completely, depending on the transmission probabilities.

## 1. Introduction

Controlling and manipulating the flow of phonons, which are the particles responsible for the transmission of heat and sound through a material, has been of great scientific and technological interest for decades [1,2,3,4,5,6,7]. Earlier efforts in this regard employed structural defects such as impurities and interfaces at the atomic scale to reduce the heat flow owing to the phonon scattering, essentially reducing the phonon mean free path [8,9,10,11,12]. However, with the recent technological advancement in terms of nanoscale device fabrication, scientists have found that nanostructuring materials with an array of specially designed pores can selectively suppress the flow of phonons with certain frequencies, as they introduce interference effects leading to the formation of phononic bandgaps in these materials [13,14,15,16]. The approach of controlling and manipulating heat transport and thermal conductivity by means of the wave interference method has an immense advantage over the phonon scattering method, as they allow heat flow control at the nanoscale level. Additionally, the range of the phononic bandgap and the desired frequency of the thermal vibrations to be suppressed can be controlled by varying the size and shape of the pores. Such materials which are engineered to control the transmission of phonons are called Phononic Crystals (PnC) and are already being used in a wide range of applications, such as noise and vibration attenuation, acoustic waveguides, and filters. By creating artificial structures combining two materials with different elasticities and periodicity from centimeter to micrometer range, frequencies in the range of KHz to MHz corresponding to sound waves have already been controlled with efficiency [16,17].

The prospects of phononic crystals to manipulate thermal conductivity and heat capacity have since then attracted researchers, and significant experimental and theoretical works have been demonstrated on silicon based phononic crystals, which is considered the most common material in electronics, micro-, and nano-electromechanical systems [18,19,20,21]. GHz phonon bandgaps were already demonstrated in silicon nitride (SiN) and silicon membranes which promotes the possibility of controlling thermal excitations in sub-kelvin temperatures and allows newer prospects in optomechanical applications [19,21,22,23,24] However, studies regarding the manipulation of room temperature phonons in the THz frequency regime are still limited, and the fabrication scheme of such nanophonon systems has only recently been explored further with the emergence of two-dimensional materials. The extraordinary properties of the 2D materials has led to the quest of novel ideas and applications in these materials [25]. Graphene, which is the first isolated two-dimensional material, is of particular interest as it showcases excellent transport properties [14,16,26]. Compared to the conventional silicon, graphene is regarded as a more promising candidate for the phononic crystals, due to the high mechanical strength with ultra-high Young’s modulus of 1 TPa. These unique characteristics of graphene make it an attractive material for nanoelectromechanical systems (NEMS) applications [27,28,29,30,31,32]. High conductivity and high surface to volume ratio due to monolayer thickness of graphene makes it a commonly explored material for sensors [33,34,35,36] and beyond complementary metal-oxide-semiconductors (CMOS) devices [37,38,39,40,41,42].

Extensive study on graphene and the development in nanofabrication techniques makes it an ideal candidate for the study of thermal properties and related applications. Although graphene has high electrical and thermal conductivity, the suppression of heat transport, keeping the high electrical conductivity, is desirable in some electronic device applications. As mentioned earlier, phononic crystals, which limit the heat flow due to the presence of an array of specially designed pores, are of utmost importance from this perspective. Recently, our group has successfully nano-patterned periodic arrays of 3–4 nm sized pores on suspended graphene using focused helium ion beam milling [43]. The robustness of the focused helium ion beam milling system allows the fabrication of more complicated nanopore patterns, such as the cross shape and hexagonal snowflake shape, with the help of a pattern generator. It was reported that circular periodic nanopores in graphene generate a phononic bandgap in the GHz regime [20,44]. The bandgap frequency is dependent on the pitch size of the pattern. With lower pitch size in the sub-10 nm regime, phononic bandgaps at higher frequencies were observed. However, for potential controllability of heat phonon propagation in thermoelectric devices, we need to obtain the phononic bandgap in the THz regime [20]. Our group previously performed theoretical simulations on circular and cross-shaped nanopores and obtained a bandgap opening in the THz regime with extremely high periodicity of the nanostructure, e.g., with neck length ≤1 nm (≈0.9 THz) [30]. In this report, we extended the study to the hexagonal lattice counterpart of the cross-shaped nano-pore, which is the ’snowflake’ shaped nanopore, to obtain a phononic bandgap at higher frequencies.

## 2. Computational Method

The calculations were carried out using the three-dimensional Finite Element Method (FEM) employed in the framework of COMSOL MULTIPHYSICS software version 5.5 [45]. To carry out the dispersion relation between the phonon energy and the wave vector, Floquet boundary conditions were used [46,47,48]. Amongst the available application-specific modules for various physics phenomena, we made use of the acoustic module to solve the wave propagation in the phononic crystal to obtain the pressure map of the system at fixed phononic frequencies. The graphene thickness was fixed to be 1 nm to favour the meshing condition.

## 3. Results and Discussion

The thermal conductivity in a material can be defined as:

(1) $$\kappa =\sum_{j}\int_{0}^{\omega_{max}}\hbar \omega_{j}\frac{\partial n_{j}}{\partial T}g_{j}v_{j}l_{j}d\omega ,$$

where *j* runs over different polarization branches of phonon, which include two transverse acoustic branches and one longitudinal acoustic branch; *ω_j_* is the phonon energy; *n_j_* is the occupation number; *T* is the temperature; *g_j_* is the density of states; *v_j_* the group velocity; and *l_j_* is the mean free path of the phonon. As far as the wave interference approach towards controlling the heat transport and thermal conductivity is concerned, various shapes of nanopores with different periods affect the dispersion relation of the phonon, which in turn affects the propagation of the phonon by changing the density of states and the group velocity. The wavelength of the phonon that was forbidden to propagate depends on the period of the nanopores as the interference effect follows Bragg’s law; *nλ* ≈ 2*a,* where *λ* is the phonon wavelength, and *a* is the periodicity of the nanopores. The shape of the phononic crystal also has an immense effect in introducing the phonon bandgap in these nanostructures.

**Figure 1:**
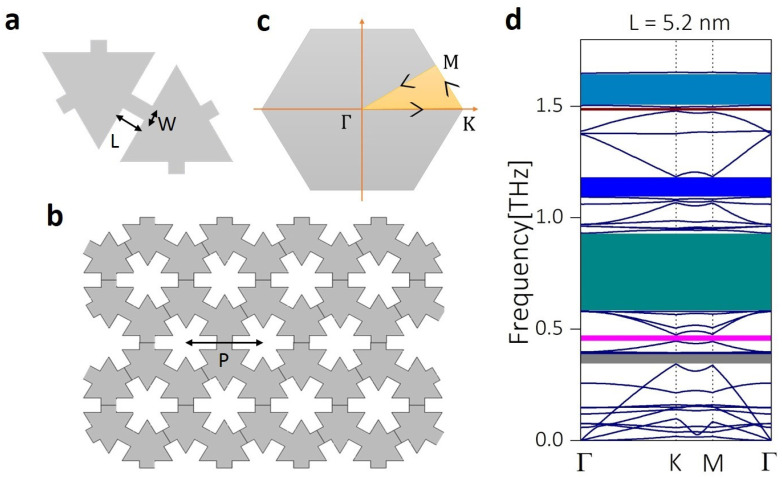
(**a**) Schematic representation of the unit cell of the hexagonal snowflake phononic crystal. L and W are the length and width of the neck of the snowflake structure. (**b**) Supercell of the snowflake phononic crystal formed when the unit cell shown in (a) is repeated in both the x and y directions. The periodicity, P, represents the distance between the centers of two snowflake nanopores and is fixed to be 25 nm throughout the calculation. (**c**) Schematic diagram showing the Brillouin zone of the hexagonal lattice. The shaded region Γ→K→M→Γ represents the path along which the phononic band structure calculation is performed. (**d**) The phononic band structure calculated for snowflake structure with neck length 5.2 nm showing the bandgaps in the THz regime which is desirable for heat phonon frequency controllability in thermoelectric devices.

Figure 1a shows the schematic representation of the unit cell of the hexagonal snowflake phononic crystal considered in this study. L and W are the length and width of the neck of the snowflake structure.

The periodic nanostructure obtained by repeating the unit cell in x and y directions is shown in Figure 1b. The periodicity of the structure is fixed to be 25 nm throughout the calculations. In this study, we focus on the variations in the interference effect as we change the neck-length of the snowflake structure. Hence, we performed phononic band structure calculation for different neck-lengths, L, along the high symmetry points of the Brillouin zone shown in Figure 1c. Figure 1d shows the band structure calculated for the snowflake structure, which has a neck length of L = 5.2 nm. A phononic bandgap at higher frequency regime (≈1.6 THz) was obtained compared to the bandgap around 0.9 THz obtained for the crossbar structure in our previous study [30]. Additionally, several other phonon bandgaps are formed at lower frequencies suppressing a large portion of the phonon frequency.

**Figure 2:**
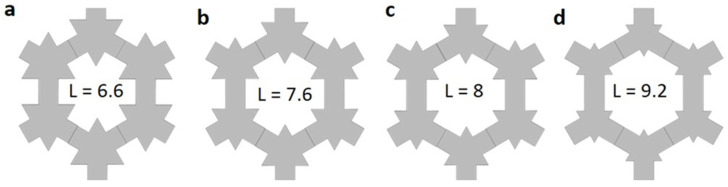
Schematic diagram showing the change in the snowflake structure with various neck lengths of (**a**) 6.6 nm, (**b**) 7.6 nm, (**c**) 8 nm, and (**d**) 9.2 nm.

**Figure 3:**
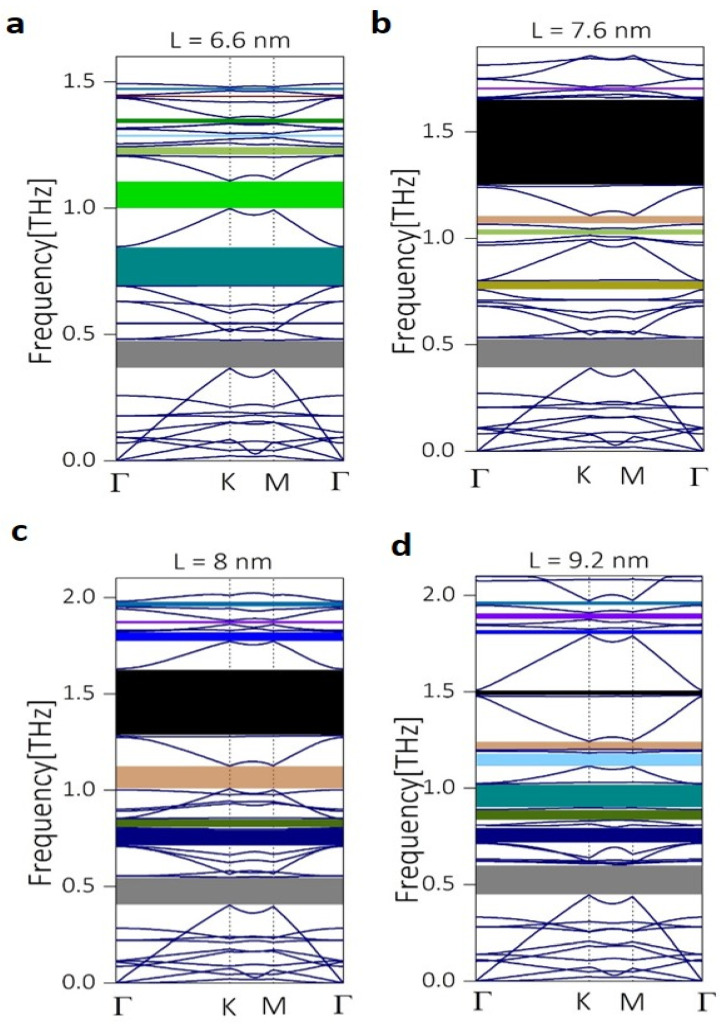
Phononic band structure calculated along the high symmetry points of the Brillouin zone for neck lengths (**a**) 6.6 nm, (**b**) 7.6 nm, (**c**) 8 nm, and (**d**) 9.2 nm of the snowflake shaped phononic crystal.

Motivated by the improvement in the phonon bandgap calculation, we carried out band structure calculations for other neck lengths. We noticed that varying the neck-length also reduced the size of the triangles in the snowflake unit cell to keep the periodicity constant (Figure 2 a–d). This could impact the dispersion relation, as the coherent interference may change with the size of the triangle due to the wave reflection from the triangular surfaces. Figure 3a–d shows the phononic band structure plotted along the high symmetry points for the neck lengths 6.6 nm, 7.6 nm, 8 nm, and 9.2 nm respectively. Compared to the large bandgap observed for L = 5.2 nm in Figure 1d, increasing the length to 6.6 nm lowered the frequency of the bandgap, and at the same time reduced the size of bandgap (Figure 3a). However, increasing the neck length further to 7.6 nm (Figure 3b) brought back the large bandgap of around 1.6 THz. Additionally, a small bandgap of a width of 14 GHz was opened around 1.7 THz (purple color). Further, increasing the neck length to 8 nm gives rise to more bands at higher phononic frequencies. Additionally, a new bandgap was opened around 1.96 THz. Note that the large bandgap present around 1.5 THz reduced in size compared to the case of L = 7.6 nm. Figure 3d shows the phononic band structure calculation for L = 9.2 nm, where the top bandgap reduced in frequency. Additionally, the large bandgap around 1.5 THz became very narrow. To summarize the observation of the bandgap opening for different neck lengths of the phononic crystal, we tabulated the range and width of the highest bandgap for all the above-mentioned neck-lengths in Table 1.

**Figure 4:**
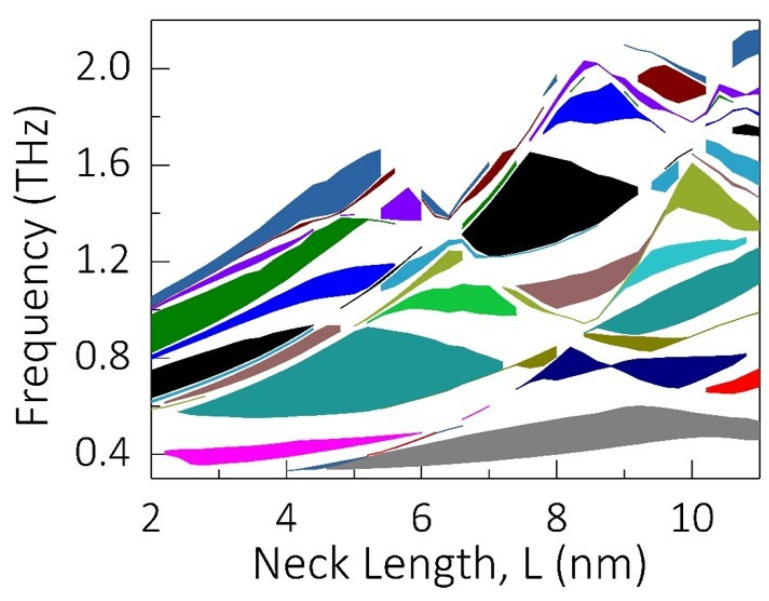
The phononic bandgap map which depicts the bandgaps plotted as a function of frequency for various neck lengths of the snowflake phononic crystal. The neck-length is continuously varied from 2 nm to 11 nm. Different colors indicate different phononic bandgaps appearing between two particular bands.

Although a steady increase in the frequency of the highest bandgap was observed until L = 8 nm, the frequency range and the width of the bandgap reduced for L = 9.2 nm. Thus, the dependence of the bandgap opening on the length of the neck is not linear as expected. This motivated us to study the phononic bandgap opening for all the possible neck lengths. Figure 4 shows the phononic bandgap map for neck length varying from 2 nm until 11 nm. The different colors indicate different bandgaps appearing between two particular bands. These colors are also matched with the colors used in Figure 1d and Figure 3 to indicate different bands. It is clear from the bandgap map that varying the neck length changes the width of the bandgap considerably. Additionally, as is evident from the bandgap map, the bandgap does not follow a linear relationship with the neck length. Moreover, some of the bands follow an oscillatory pattern, where the bandgap width increases with the length, reaches a maximum, and then decreases with increase in neck-length. Such a pattern can be observed especially in the case of the large hill shaped bands centering at 5 nm and 8 nm neck length (cyan and black respectively). Additionally, it is noteworthy that the overall bandgap map has an oscillatory pattern which peaks at neck lengths of 5.4 nm and 8.4 nm. Such a bandgap map will help to selectively choose the parameters of the snowflake phononic crystal to suppress the thermal conductivity in the THz regime.

**Figure 5:**
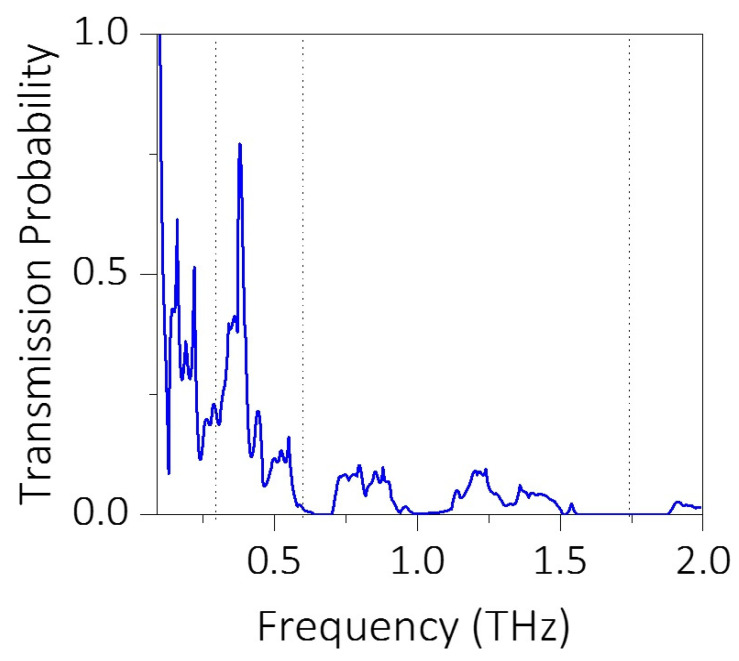
The transmission probability calculated as a function of phonon frequency for the snowflake shaped phononic crystal having neck length of 8 nm. The portion of the spectrum with zero transmission probability represents the phononic bandgaps in the band structure calculation.

We also calculated the transmission probability as a function of phonon frequency for the snowflake shaped phononic crystal with a neck length of 8 nm (Figure 5). The transmission of the longitudinal waves was reported for a two-dimensional phononic crystal system with a 10 × 1 nanopore arrangement. We performed frequency domain analysis in solid mechanics to obtain time-harmonic analysis of acoustic wave propagation in the system using the following equation:

(2) $$\frac{1}{\rho_{0}c^{2}}\cdot \frac{\partial^{2}p}{\partial t^{2}}+\left(-\frac{1}{\rho_{0}}\nabla p\right)=0.$$

Here, p is the pressure, c is the speed of sound in the medium, and ρ_0_ is the density. This equation can be reduced to a Helmholtz relationship for a time harmonic pressure wave excitation, as expressed by the following equation:

(3) $$\nabla \left(-\frac{1}{\rho_{0}}\nabla p\right)-\frac{\omega^{2}p}{\rho_{0}c^{2}}=0,$$

Here, ω is the angular frequency defined by ω = 2πf. Equation (3) can be solved to obtain the pressure field, and using a parametric solver, the corresponding frequency spectrum was constructed [48].

**Figure 6:**
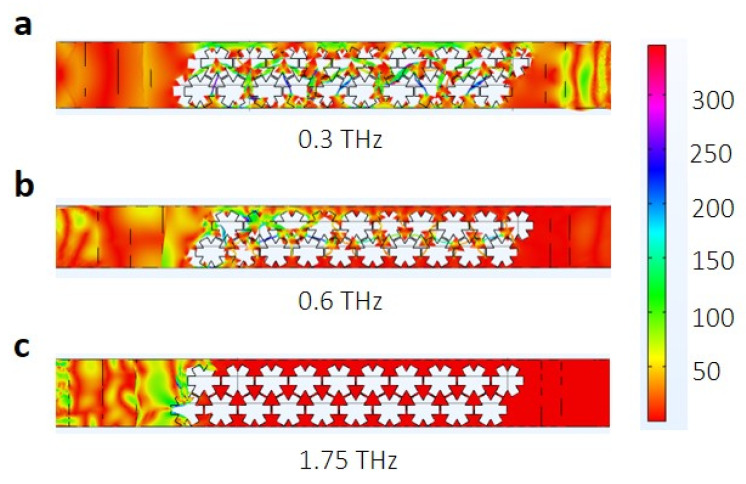
The transmission map, which analyses the transmission of the phonons with a particular frequency through the snowflake phononic crystal. The transmission characteristics for phononic frequencies of 0.3 THz, 0.6 THz, and 1.75 THz are shown in (**a**), (**b**) and (**c**) respectively. High transmission probability is observed for 0.3 THz waves. For 0.6 THz, the transmission probability is reduced significantly while for 1.75 THz, a complete phonon blockade is observed.

Complete suppression of frequencies around 0.7 THz, 1 THz, and 1.75 THz observed in the transmission probability spectrum represents the phononic bandgaps. When compared with the phononic band structure calculated for the snowflake structure with neck length 8 nm (Figure 2c), an upward shift of 0.27 THz in the phonon frequency was observed in the transmission spectrum. Such a difference is expected due to the limited number of nanopores used in the transmission spectrum analysis (Figure 5). Additionally, the diffraction effects from the two edges of the snowflake structure would have contributed to this anomaly. Nonetheless, apart from this upward shift in the frequency, the position of the bandgap and the width of the bandgap is in good agreement with the band structure calculation shown in Figure 3c. To confirm the propagation of phonons with certain frequency through the phononic crystal, we visualized the transmission characteristics for various frequencies in the transmission spectrum. Here, an acoustic wave of a certain frequency is applied at the left end of the sample and the transmission of the wave through the phononic crystal was visualized. The transmission of the acoustic wave for 0.3 THz, 0.6 THz, and 1.75 THz are shown in Figure 6a–c respectively. As for 0.3 THz, which shows high transmission probability, most parts of the wave reached the other end of the phononic crystal as expected. For 0.6 THz frequency, which has a very small transmission probability, a small portion of the wave reached the other end. However, for the 1.75 THz wave, which falls in the bandgap region of the transmission spectrum, the wave is completely blocked by the phononic structure, which substantiates the ability of the snowflake nanopores in suppressing thermal conductivity.

As we saw, the shape of the nanopores have an immense effect on the phonon dispersion relation as well as the transmission probability. We performed resultant thermal conductivity calculations, in order to understand the effect of the nanopore shapes on the thermal properties in graphene-based materials. The thermal conductivity is calculated by:

(4) $$\rho \mathrm{Cp}u\;.\;\nabla T-\nabla \cdot \left(\kappa \nabla T\right)=Q+Q_{\mathrm{ted}}.$$

**Figure 7:**
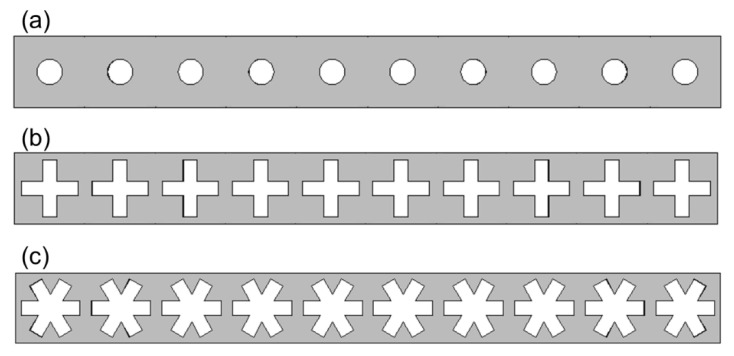
Supercell of the (**a**) circle, (**b**) cross, and (**c**) snowflake shaped graphene phononic crystal used for the thermal conductivity simulation.

Here, ρ is the density of the material, C_p_ is the specific heat capacity at constant pressure, **u** is the displacement field, −k ∇T is the conductive heat flux where ∇T is the temperature difference, Q is the heat source, and Q_ted_ is the thermoelastic damping. The resultant thermal conductivity for snowflake-shaped phononic crystal was calculated and compared to that of circular and cross-shaped PnCs [49]. Figure 7a–c shows the schematic diagram of the circular, cross-shaped, and the snowflake-shaped PnCs, respectively. To be consistent with the circle and cross-shaped nanopore supercell boundary conditions, similar boundary conditions in horizontal and vertical directions were used for snowflake shaped nanopores as well. We observed that the usage of hexagonal boundary conditions for snowflake shaped graphene PnCs led to relatively lower thermal conductivities in snowflake shaped PnCs. A square unit cell of size 25 nm was used for each of these nanopores. This unit cell was repeated 10 times in the horizontal direction, which led to a supercell of length 250 nm and width 25 nm, as shown in Figure 5. Previous reports suggested that the thermal conductivity increases with length up to 250 nm of the PnC, and then decreases slowly with increasing length [44]. The material parameter, thermal conductivity of graphene, was taken as 3000 W/(m·K) [50].

All the PnCs show a reduction in the thermal conductivity as a function of porosity. However, circular pored PnC shows a large reduction in the thermal conductivity with the increase in porosity compared to the other two PnC structures.

**Figure 8:**
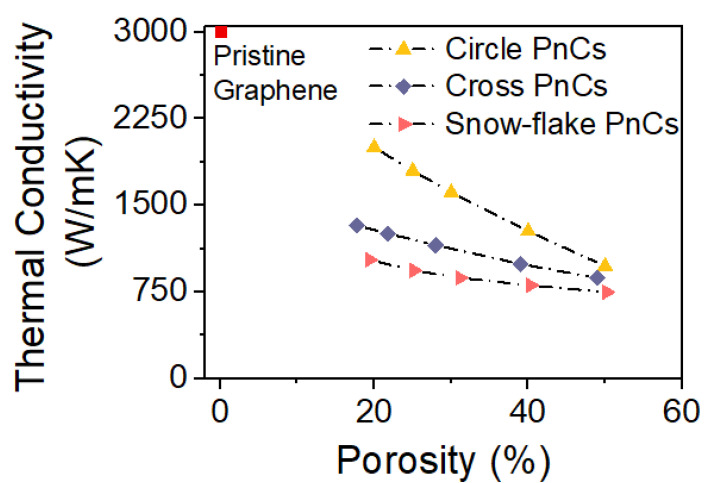
Shows the simulated thermal conductivities for the supercell structures shown in Figure 7 for different porosities. The radius of the circle is varied for circular shaped PnCs and width of the rectangular pore is varied for cross and snowflake shaped pores. For comparison, graphene thermal conductivity (3000 W/(m·K)) is shown as a point in the plot.

The change in thermal conductivity for different PnCs (circular, cross and snowflake) with same porosity implies that the shape of the nanopore indeed has an effect on the thermal conductivity (Figure 8). This difference in thermal conductivity, which is a geometrical effect, comes from the difference in cross sectional area through which the heat can propagate. The larger reduction in thermal conductivity as a function of porosity for circular PnC indicates that the reduction in the cross sectional area through which the heat can propagate is larger when the radius of the circle is increased. However, for cross and snowflake-shaped PnCs, such volume reduction effect is smaller with the increase in neck-width. It is also noteworthy that the thermal conductivity for all the PnCs become almost equal around 50% porosity [50]. This can be explained as follows. Since the unit cell size of 25 nm is kept constant for all the PnCs, the porosity is increased by increasing the radius of the circle for circular pored PnC, while for cross and snowflake-shaped PnCs, the width of the neck is increased. Thus when the neck width is very large, the cross and snowflake pores resemble that of a circle. This result in similar thermal conductivity at higher porosity for all the PnCs.

## 4. Conclusions

In conclusion, we have investigated thermal conductivity regulation in snowflake shaped graphene phononic crystals. As a result of the coherent interference from the snowflake nanopores, a phononic bandgap in the THz regime was obtained, which is important for heat phonon controllability in thermoelectric devices. The size of the bandgap and its position in the phonon dispersion curve could be manipulated by varying the neck length of the snowflake structure. A distinctive bandgap map was also computed by varying the neck length of the snowflake structure, which provides enormous amounts of information as to the size and position of the phononic bandgap at various neck lengths. The transmission probability calculation as a function of phonon frequency also shows good agreement with the band structure calculation. The pressure map of the phononic crystal for various frequencies having different transmission probability also validate the effectiveness of snowflake shaped nanopores in suppressing the phonons with frequencies in the bandgap region.

## Figures and Tables

**Table 1:** The range and the width of the highest bandgap for all the neck lengths considered in the Figure 3.

L	Phononic Bandgap Range	Phononic Bandgap Width
(nm)	(THz)	(GHz)
6.6	1.465–1.479	14
7.6	1.698–1.711	13
8	1.951–1.980	29
9.2	1.948–1.971	23
